# A chemistry-informed deep learning network for mitigating the stratospheric OH data gap

**DOI:** 10.1126/sciadv.aee2319

**Published:** 2026-07-08

**Authors:** Wenjie Yin, Chen Zhou, Wuhu Feng, Sandip S. Dhomse, Richard J. Pope, Luis Millán, Martyn P. Chipperfield

**Affiliations:** ^1^ School of Earth and Space Science Technology, Wuhan University, Wuhan, China.; ^2^ School of Earth and Environment, University of Leeds, Leeds, UK.; ^3^ National Centre for Atmospheric Science, University of Leeds, Leeds, UK.; ^4^ National Centre for Earth Observation, University of Leeds, Leeds, UK.; ^5^ Jet Propulsion Laboratory, California Institute of Technology, Pasadena, CA, USA.

## Abstract

The hydroxyl (OH) radical is a key species in stratospheric chemistry, driving ozone loss and the coupling of other chemical families. However, global OH observations are very sparse, with a critical gap in satellite-based observations since 2009. Although alternative chemical methods attempt to mitigate such gaps, they have limitations. Here, we present DRCAT, a chemistry-informed deep learning network to predict stratospheric OH profiles using coincident satellite observations of chemically related species. Trained on only 2 years of data, DRCAT provides reconstruction of a continuous and reliable global stratospheric OH dataset from late 2004 to the present. Results show that DRCAT outperforms both a chemical transport model and an observation-constrained steady-state approximation. Moreover, DRCAT successfully predicts anomalous OH enhancements after the 2022 Hunga volcanic eruption, showing generalizability to extreme conditions outside of the training regime. DRCAT thus provides a robust pathway to fill OH observational voids. Beyond OH, this work offers a scalable framework for similar reconstruction of other key short-lived species.

## Teaser

A chemistry-informed deep learning model reconstructs a continuous satellite-based global stratospheric OH record.

## INTRODUCTION

The hydroxyl (OH) radical plays an important role in many major chemical processes in the stratosphere. OH participates in the HOx (= OH + HO_2_ + H) catalytic cycles that destroy ozone in the upper and lower stratosphere and plays a central role in the interconversion of radical and reservoir species for other chemical families. Accurate measurements of the spatial and temporal variability of OH are therefore important. However, because of its short lifetime (~1 s) and low concentration (~10^6^ molecules cm^−3^), measurements of OH are sparse, especially in the stratosphere. In situ observations have been made from limited balloon (*1*) and aircraft (*2*) flights. Remote sensing observations in the far-infrared have also been made from balloon flights (*3*, *4*). Long-term ground-based remote sensing column observations allow for the inference of OH variability in the upper stratosphere and mesosphere (*5*, *6*). Although these measurement techniques offer ongoing localized observations, they suffer from inherent limitations in spatial and temporal coverage.

Global observations of OH are highly desirable. The Aura Microwave Limb Sounder (MLS) (*7*, *8*) provided daily global OH measurements from late 2004 through the end of 2009, which have been extensively validated (*9*–*11*). After more than 5 years of nearly continuous operation, the OH subsystem began to degrade and was deactivated to preserve its remaining lifetime. From 2011 to 2014, the subsystem was reactivated for about 30 days each August to capture data spanning the 11-year solar cycle.

In the absence of direct global measurements, alternative methods have been developed to infer OH variations. It has been possible to infer the global mean OH concentration using top-down approaches through budget closure or inversion of measurements from long-lived trace gases, such as methyl chloroform (*12*), but these values lack spatial resolution and are clearly heavily weighted to the troposphere.

Chemical transport models (CTMs) simulate stratospheric OH distributions by integrating comprehensive chemical reaction kinetics and meteorological information of temperature and transport processes within a three-dimensional (3D) framework. However, the accuracy of these model calculations is limited by uncertainties in reaction mechanisms and photochemical parameters, along with the modeled abundance of other reactant species and the accuracy of transport processes. Given its many chemical interactions, and fast photochemistry, spatially resolved estimates of OH can be obtained from modeled values of other chemical tracers and assuming the photochemical steady-state approximation (SSA) between sources and sinks. The accuracy of the SSA method depends partly on the number of source and sink terms, as well as the photochemical data. An extension of this model-based SSA approach is to combine the photochemical theory with observations of other reactant species, such as the coupling of HNO_3_ and NO_2_ (*13*) or a more complete analysis of all major OH source and sink reactions (*14*). This approach will overcome model uncertainties in the abundance of trace gases, but it will still depend on laboratory-determined parameters and model-based calculation of photolysis rates.

Recently, machine learning (ML) has emerged as a viable way to infer tropospheric OH. Some studies used tree-based ML algorithms (e.g., XGBoost) to obtain near-surface OH concentrations or column values by using a set of satellite-observed proxy variables (*15*–*17*). These studies established the feasibility to infer OH by combing ML and a set of chemical drivers to infer OH. However, they are constrained by the lack of direct tropospheric OH observations, forcing a reliance on model-simulated OH as training targets.

With evolving algorithms and increasingly comprehensive datasets, deep learning offers a promising and powerful pathway to leverage the wealth of information in historical satellite observations beyond that possible with existing methods (*18*–*21*). In this study, we introduce DRCAT (deep learning–based radical species chemistry-aware tracer), a chemistry-informed deep learning network for predicting radical concentrations. We apply it to stratospheric OH, using vertical MLS profiles of related species as inputs to infer OH concentrations and uncertainties. We use a hybrid deep learning architecture that combines graph neural networks (GNNs) and transformers to process the vertical profiles and chemical interactions (*22*–*24*). Central to DRCAT is an innovative chemistry-informed two-stage training scheme. DRCAT is first pretrained on an enhanced SSA dataset to learn fundamental chemical knowledge and then fine-tuned on MLS observations to correct biases, ensuring that predictions are both data-driven and physically consistent. DRCAT successfully reproduces realistic, smoothed OH profiles and outperforms other baseline methods among various metrics. Results demonstrate that this method provides a continuous, reliable, and physically consistent dataset for stratospheric OH throughout the MLS mission. Validation results after the 2022 Hunga volcanic eruption further reveal its ability to handle extreme conditions, providing a robust pathway to fill OH observational gaps and reevaluate the chemical states of the atmosphere even during periods of substantial perturbations.

## RESULTS

To evaluate the model robustness fairly, we trained DRCAT on 2 years of MLS data (2005–2006) and validated its performance using observations from 2007 to 2009, spanning the MLS OH operational period.

### Model performance assessment

**Fig. 1.**
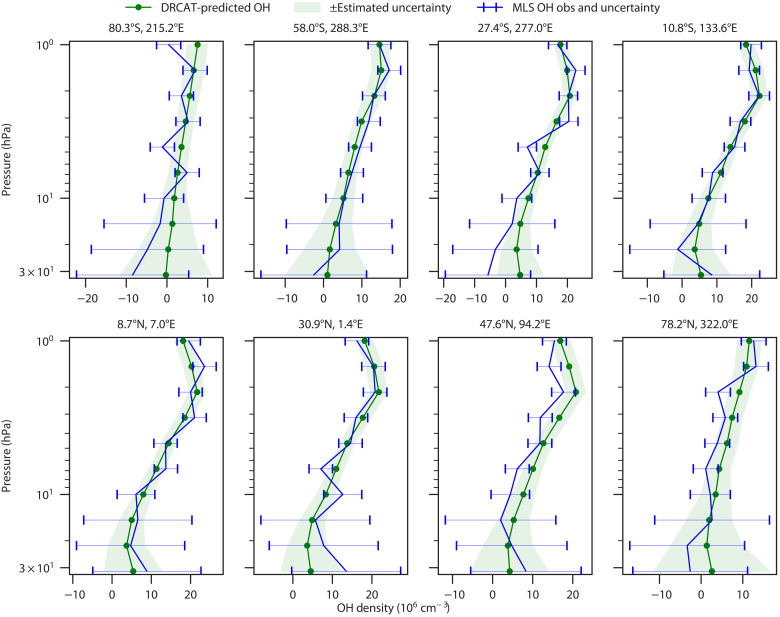
Comparisons of vertical OH profiles. Comparison of MLS OH observational profiles (molecules cm^−3^) and total uncertainty (blue) with DRCAT predictions and estimated ±1σ uncertainty (green) for eight randomly selected samples on 10 February 2008 plotted from south to north. The profile coordinates are indicated in the panel titles.

As an illustrative example, the comparison of DRCAT’s predicted OH vertical profiles with observed MLS OH profiles is shown in Fig. 1 and fig. S1. The DRCAT predictions (green line) closely track the observed MLS OH (blue line) across a wide range of pressure levels, consistently staying within the predicted uncertainty bounds (green shading). The blue error bars represent the overall uncertainty of the MLS observations, calculated from the root sum square of the instrument’s documented accuracy and precision at different pressure levels (*25*). The observed MLS profile exhibits “zigzag” variations or even yields negative values, particularly in the range of 32 to 14 hPa, due to the large precision uncertainty of the MLS data in this region. Notably, DRCAT captures the underlying vertical distribution and effectively smooths the MLS observations, producing physically plausible OH profiles.

**Fig. 2.**
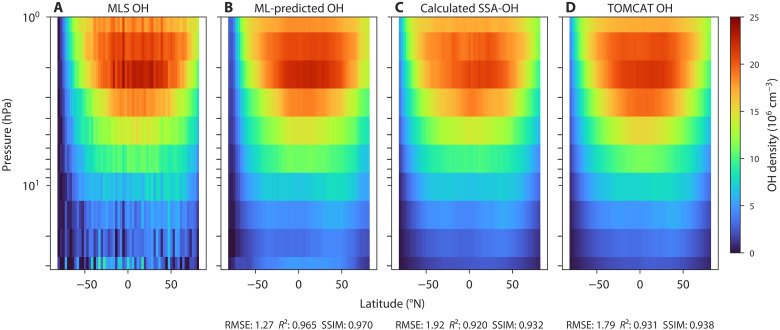
Comparisons of global daily zonal mean OH for four data sources on 10 April 2007. (**A**) MLS OH observations. (**B**) DRCAT-predicted OH. (**C**) Calculated SSA-OH. (**D**) TOMCAT CTM simulation.

Given the inherent noise in the single vertical profile, by taking a zonal average of the global data, we can better observe the overall trends in OH distribution and assess the model’s performance. On a global scale, the model accurately captures the zonal mean distribution of OH (Fig. 2 and fig. S2). For example, compared to the MLS OH observations, the global zonal OH concentrations predicted by DRCAT show a high degree of consistency in both OH magnitude and morphology, whereas biases can be seen in the SSA-OH and TOMCAT-OH results in certain areas. In addition, DRCAT’s predicted OH concentrations show excellent agreement with the near-zero OH values observed at the poles, which is due to minimal photochemical reactions for OH production in polar winter. These are clearly reflected by the performance metrics. On this day, DRCAT achieves a root mean square error (RMSE) of 1.27 × 10^6^ molecules cm^−3^, a spatial correlation (*R*^2^) of 0.965, and a structural similarity index (SSIM) of 0.97. This performance is a substantial improvement over both the calculated SSA-OH (RMSE = 1.92 × 10^6^ molecules cm^−3^, *R*^2^ = 0.92, and SSIM = 0.932) and the simulated TOMCAT-OH (RMSE = 1.79 × 10^6^ molecules cm^−3^, *R*^2^ = 0.931, and SSIM = 0.936).

**Fig. 3.**
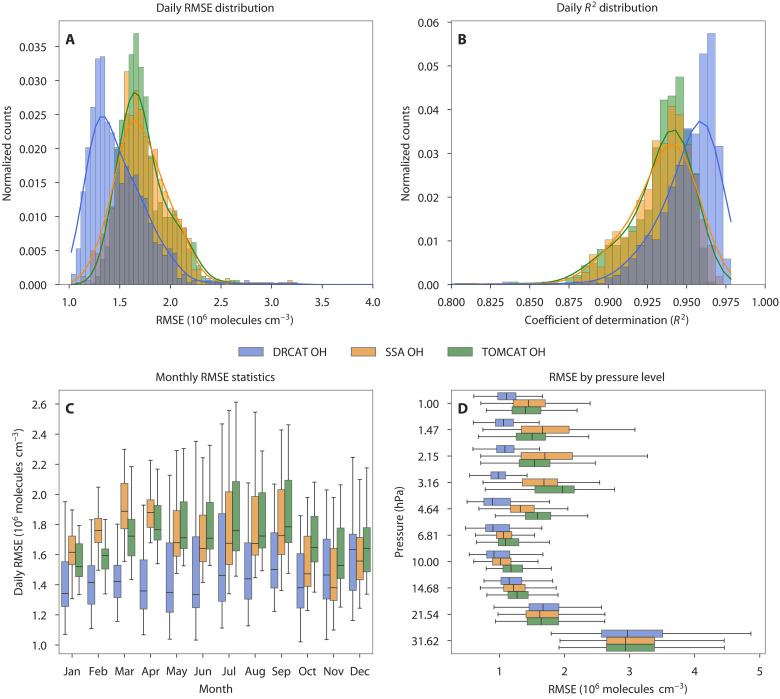
Statistical evaluation of model performance. (**A** and **B**) Normalized histograms of daily RMSE (molecules cm^−3^) and *R*^2^ values for DRCAT (blue), calculated SSA-OH (yellow), and simulated TOMCAT OH (green) compared with MLS observations. (**C** and **D**) RMSE statistics by month and by pressure level for three methods compared with MLS observations.

Statistical analysis of all MLS OH data (excluding training period) further confirms the superior performance of DRCAT compared with SSA and TOMCAT (Fig. 3). The distribution of daily RMSE (lower is better) for DRCAT is centered at a smaller value (~1.25 × 10^6^ molecules cm^−3^), outperforming the broader and higher error distributions of the SSA and TOMCAT models (Fig. 3A). Similarly, the daily *R*^2^ (higher is better) distribution for DRCAT is clustered near 0.97, indicating consistently high correlations with MLS observations (Fig. 3B). An analysis of RMSE by month and pressure level reveals that DRCAT maintains low error throughout the stratosphere at all times, whereas the SSA and TOMCAT methods exhibit large errors, particularly in the upper stratosphere where OH concentrations are greater (Fig. 3, C and D).

In fig. S3, we further analyze the OH diurnal variation as a function of solar zenith angle (SZA) at the 2.15-hPa pressure level (the OH stratospheric peak) during 2008. As shown in fig. S3A, MLS OH measurements exhibit a wide spread of values at any given SZA, due to large random precision uncertainties. In contrast, DRCAT OH displays a more compact distribution. We apply a two-parameter exponential fit for all datasets, following the equation used by Minschwaner *et al.* (*26*). The fitted curves (fig. S3E) demonstrate good agreement between DRCAT and the MLS observations. Specifically, coefficient β for DRCAT (0.326) is consistent with that for MLS OH (0.322), indicating that DRCAT effectively captures the underlying solar-driven photochemical relations.

### Predicting OH variations during Hunga eruption

Although DRCAT performs well under normal atmospheric conditions, a major question is its robustness under extreme, out-of-distribution conditions. The January 2022 Hunga eruption injected an unprecedented ~150 Tg of water vapor directly into the stratosphere, resulting in notable and long-lasting stratospheric perturbations (*27*–*29*). This event provides a natural test of DRCAT’s generalization ability under extreme conditions.

**Fig. 4.**
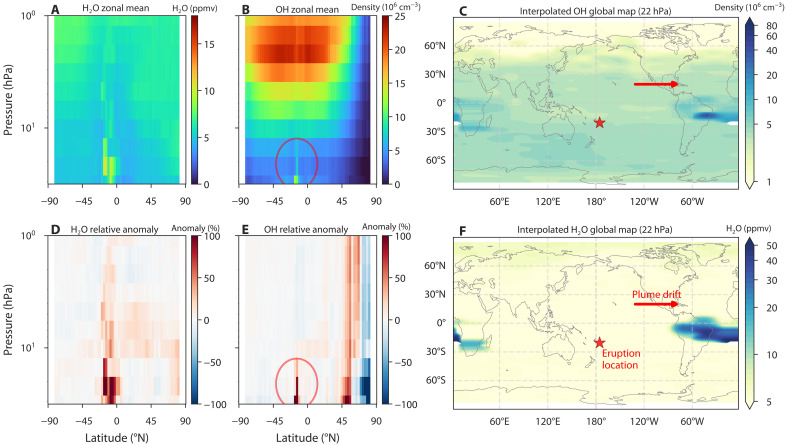
MLS H_2_O and DRCAT-predicted OH variations during Hunga eruption on 25 January 2022. (**A** and **D**) Zonal mean (ppmv) and relative anomaly (%, 2022 versus 2021) values of MLS H_2_O. (**B** and **E**) Zonal mean (molecules cm^−3^) and relative anomaly (%, 2022 versus 2021) values of DRCAT-predicted OH. (**C** and **F**) DRCAT-predicted OH and MLS H_2_O interpolated global maps at 22 hPa.

**Fig. 5.**
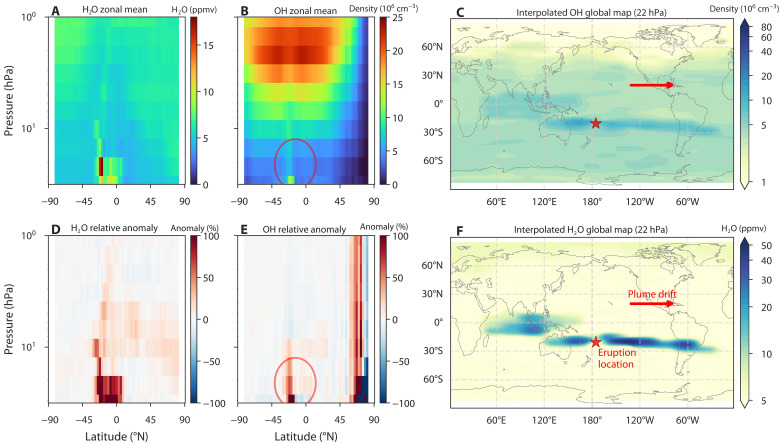
MLS H_2_O and DRCAT-predicted OH variations during Hunga eruption on 3 February 2022. (**A** and **D**) Zonal mean (ppmv) and relative anomaly (%, 2022 versus 2021) values of MLS H_2_O. (**B** and **E**) Zonal mean (molecules cm^−3^) and relative anomaly (%, 2022 versus 2021) values of DRCAT-predicted OH. (**C** and **F**) DRCAT-predicted OH and MLS H_2_O interpolated global maps at 22 hPa.

Figure 4 illustrates the substantial impact of the volcanic eruption on stratospheric H_2_O and the corresponding response in OH predicted by DRCAT. Figure 4A clearly shows a marked increase in stratospheric water vapor, particularly in the low- to mid-latitudes of the Southern Hemisphere following the eruption. Specifically, absolute H_2_O concentrations increase from typical background values of 4 to 5 parts per million volume (ppmv) to over 10 ppmv in the region of 32 to 10 hPa (0°S to 30°S). The relative anomaly plot (Fig. 4D), comparing 2022 to 2021, highlights these enhancements more distinctly, with peak relative anomalies exceeding 100%. Correspondingly, DRCAT predictions of global OH concentrations (Fig. 4, B, C, and E) reveal clear enhancements in regions affected by the H_2_O enhancement. Because of the influence of global circulation and volcanic plume drift, the injected water vapor at the 22-hPa level is not centered over the eruption site on 25 January. As the plume drifts over subsequent days (Fig. 5F), the interpolated global map of DRCAT-predicted OH at 22 hPa (Fig. 5C) exhibits corresponding enhancements in the Southern Hemisphere low latitudes (0°S to 30°S). This demonstrates the model’s ability to capture the dynamic evolution of chemically coupled perturbations.

Although there are no direct observations to provide OH validation during Hunga eruption, the OH enhancement is widely qualitatively recognized, as evidenced by numerical simulations showing substantial increases in OH following volcanic injections (*28*–*30*). Wilmouth *et al.* (*28*) reported a substantial increase in HOx (OH, HO_2_, and H), and the modeled rate of the O(^1^D) + H_2_O reaction increases by approximately a factor of 2 at the water vapor peak relative to the runs with no volcanic eruption. In addition, the calculated SSA-OH following the Hunga eruption also shows enhancements of similar magnitudes along with the increase of water vapor (see figs. S4 to S6).

Although large percentage anomalies (Figs. 4E and 5E) are also visible at high northern latitudes (>50°N), these are artifacts unrelated to the eruption. The global OH distribution is dominated by photochemistry, causing concentrations to drop to near zero in the polar night. This solar-driven pattern shifts daily with Earth’s orbit. Consequently, a slight spatial misalignment of the near-zero regions could produce large anomaly value. This can also be observed in the SSA-calculated OH relative anomaly plots in fig. S6.

DRCAT qualitatively predicts the anomalous OH enhancement following the Hunga eruption. Models trained solely on historical data from quiescent years are not equipped to handle such extreme conditions because the established relationships between OH and its predictors were fundamentally altered. DRCAT’s capability stems directly from its chemistry-informed training strategy (see Materials and Methods for details). During pretraining, the model is trained on a theoretically derived SSA dataset augmented with random perturbations, which simulates diverse atmospheric states. The physical consistency enforced during pretraining prevents the model from being constrained by historical correlations, allowing it to extrapolate on the basis of learned chemical principles. Although these results suggest that extrapolation to extreme events is possible, further validation is required to determine the quantitative accuracy. Nevertheless, this demonstrates the model’s utility not only for gap filling but also as a tool for research and hazard assessment in the face of previously unknown atmospheric phenomena.

### Smoothing capability of DRCAT

MLS OH retrievals are hindered by measurement noise, which introduces irregular fluctuations (zigzag patterns) and negative values in regions with poor signal-to-noise ratio, complicating the accurate interpretation of underlying chemical signals (*25*). From the comparisons in Fig. 1, the DRCAT’s outputs effectively reduce fluctuations while maintaining alignment with MLS-observed OH. This is further confirmed by DRCAT’s OH results from 2011 to 2014 (see figs. S7 and S8). During this period late in the instrument’s lifetime, the MLS OH observations exhibit notably larger noise and frequent data gaps. DRCAT effectively mitigates these issues, successfully generating smoothed, physically plausible OH estimates while faithfully preserving the core patterns and magnitudes of the MLS observations. Furthermore, DRCAT demonstrates a capability to reconstruct OH concentrations when MLS OH observations are missing.

Trained on 2 years of MLS data and validated against observations from 2007 to 2009, DRCAT demonstrates strong smoothing capability. Expanding the training to cover the full 2005 to 2009 dataset would not only improve predictive accuracy but also yield a comprehensive, cleaned OH dataset. The DRCAT dataset holds independent value for downstream atmospheric research, such as analyzing long-term stratospheric OH trends and evaluating CTMs. Furthermore, the DRCAT method holds promising potential for application to other MLS radical species (e.g., BrO, HOCl, and HO_2_) that are similarly affected by noisy measurements.

Notably, a common limitation of ML models is the tendency to produce overly smoothed outputs such as global weather forecasting models (*31*, *32*). This smoothing effect can obscure the accurate representation of high-variability phenomena, like extreme weather events. In contrast, for OH prediction, where the MLS OH serving as training targets are inherently noisy, this smoothing characteristic therefore becomes advantageous.

### Driving factors of OH variational pattern and shortcut learning

The selection of input features for DRCAT is guided by established chemistry knowledge and determined through a series of experiments (see section S3 for full details). The chosen features (H_2_O, O_3_, temperature, HNO_3_, N_2_O, HCl, and HO_2_) are known to be chemically linked to OH production and loss.

Notably, temporal and spatial variables (time, latitude, longitude, and SZA) are excluded from the DRCAT inputs, which is different from conventional ML practice in atmospheric science (*17*, *33*, *34*). This means DRCAT is a purely chemistry-driven model, relying solely on vertical profiles of chemical species to infer OH concentrations. This chemistry-driven approach is further validated by the model’s ability to capture solar-driven OH global variability, which shifts longitudinally, following the diurnal cycle of solar irradiance due to Earth’s orbit. DRCAT can well produce near-zero OH values in wintertime polar regions where photochemical reactions are minimal. This indicates that DRCAT effectively infers the absence of solar irradiance through learned photochemical relationships without explicit SZA information.

During model development, we observed that ML models trained exclusively on spatiotemporal features could achieve high accuracy for the OH dataset during 2004 to 2009. This phenomenon arises probably because, under normal atmospheric conditions, OH variations are predominantly governed by solar-driven processes, wherein OH production is primarily controlled by the photolysis of ozone (*26*, *35*–*37*). Consequently, such ML models learn a simplistic “shortcut” by correlating OH patterns with predictable solar cycles, rather than capturing the underlying chemical dynamics (*38*). In addition, this shortcut leads to critical failure in extreme scenarios like the Hunga eruption, where the massive injection of H_2_O strongly perturbed OH spatiotemporal patterns. Shortcut learning in the ML model would lead to drastic underestimations of the OH anomaly because it fails to learn the underlying chemistry. Our investigation indicates that this shortcut learning is too strong during model training, even when applying modern ML regularization techniques such as dropout and weight decay (*39*, *40*). Therefore, spatiotemporal variables are removed. This also motivates the adoption of chemistry-informed design and training scheme, including the pretraining on SSA-calculated dataset augmented with perturbations to simulate diverse atmospheric conditions and improve robustness. This shortcut learning underscores the necessity of chemistry-informed architectures, which prioritize physical consistency to enhance model generalizability under extreme conditions.

## DISCUSSION

Here, we have presented DRCAT, a chemistry-informed deep learning model to predict stratospheric radical profiles, with estimated uncertainties, and applied it to stratospheric OH using MLS observations. DRCAT reconstructs stratospheric OH from MLS observed species and produces physically consistent and accurate OH profiles with lower errors continuously and globally. Trained on 2 years of MLS data, DRCAT demonstrates very good performance under comprehensive evaluation. It provides smoother OH profiles that do not exhibit the zigzag variations seen in the MLS OH measurements. Validated on a global scale using a suite of metrics, DRCAT outperforms other methods, including the TOMCAT CTM and the observation-constrained SSA method. Furthermore, we validated DRCAT’s generalizability through the extreme event of the 2022 Hunga eruption. Despite the unprecedented injection of water vapor into the stratosphere, DRCAT qualitatively predicted plausible OH enhancements, demonstrating its robustness and its capacity to extrapolate beyond its training data based on learned chemical relationship.

Although DRCAT yields very promising results, certain limitations should be noted. First, the absence of direct post-2009 OH observations restrict validation, potentially limiting confidence in DRCAT’s long-term extrapolation. This challenge can be partially mitigated by cautiously using the limited MLS OH data available for several months between 2011 and 2014. If new data sources become available, they will be prioritized for further validating the DRCAT product. Furthermore, DRCAT’s application here using MLS-observed species only omits key contributors like CH_4_, which plays an important role in HOx chemistry and could enhance predictive accuracy if incorporated (*41*, *42*). In addition, the generated DRCAT OH dataset, although valuable, requires ongoing community involvement for validation to ensure its robustness.

DRCAT holds substantial promise for improvements. Extending DRCAT to predict or smooth other short-lived radicals and applying to other available satellite products (e.g., ACE-FTS) could provide a more comprehensive stratospheric chemistry application. The continuous, global OH dataset produced by DRCAT offers broad scientific utility, beyond that of the MLS OH dataset alone, facilitating insights into ozone depletion cycles and stratospheric responses to volcanic eruptions and anthropogenic influences. Last, given the aging MLS satellite and the reliance of our model on its high-quality observations (*43*), this study strongly underscores the critical scientific need for an MLS successor to ensure the continued monitoring of stratospheric OH.

## MATERIALS AND METHODS

### Dataset preparation

MLS data are available from late 2004 to the present, whereas OH is only available until 2009, with only a few months of measurements available during 2011 to 2014. We use MLS Level 2 version 5 data, applying the quality screening described by Livesey *et al.* (*25*). The day-night OH bias correction is used at the 21- and 32-hPa pressure levels as suggested. Then, we mapped the MLS data onto the TOMCAT model latitude-longitude grid with a resolution of ~2.8°, which helps reduce the measurement noise and allowed direct comparisons. We select data within pressure levels from 32 hPa (MLS OH lowest pressure range) to 1 hPa (stratopause) to focus on stratosphere. To preserve observational characteristics, the MLS data were not interpolated vertically. The TOMCAT vertical grid was interpolated to the specific pressure levels of the MLS data.

A simulation of the TOMCAT 3D CTM (*44*) was performed using the standard stratospheric chemistry scheme and ERA5 meteorological reanalysis [e.g., see Chrysanthou *et al.* (*45*)]. The model has a detailed description of stratospheric Ox, HOx, NOy, Cly, and Bry chemistry and a comprehensive selection of source gases. In addition, TOMCAT also includes time-varying solar irradiances and stratospheric sulfate area density as described in previous studies (*46*, *47*). The model calculates HOx species concentrations (H, OH, and HO_2_) by solving the full steady-state equations (based on all relevant model reactions) for each species in an iterative and coupled way. The 3D simulation was integrated from 1979 to the present day at a horizontal resolution of 2.8° × 2.8° and 32 levels from the surface to around 60 km. Model output, including photolysis rates, was sampled daily at 1:30 p.m. to coincide with the MLS observation time.

For the SSA OH dataset, we calculate OH on the basis of the SSA method using TOMCAT simulated photolysis data and MLS observations as constraints for the years of 2004 to 2024 (*48*). Notably, the SSA-enhanced dataset is only used for DRCAT pretraining. To simulate a larger range of atmospheric conditions, we add random perturbations to MLS observations and recalculate OH on the basis of SSA equations to enhance the dataset. Specifically, these perturbations are constrained within 0.5 SDs of their historical annual variability. We determined this empirical value through testing to achieve a balance. It introduces sufficient variability to aid the learning but avoids overly large perturbations that would generate excessive unphysical atmospheric states and degrade the training performance.

### Deep learning model architecture

DRCAT is based on deep learning (see fig. S9). Here, the model infers OH and its uncertainties using vertical MLS profiles of key chemical species as input features, including H_2_O, O_3_, temperature, HNO_3_, N_2_O, HCl, and HO_2_. The DRCAT model uses a hybrid architecture that integrates a GNN layer with transformer layers to effectively capture dependencies across pressure levels and extract information from chemical variables. Inspired from series-aware frameworks for multivariate time-series forecasting (*24*), this hybrid design treats pressure levels as nodes in a graph, enabling the modeling of interlevel relationships while shared transformer layers learn intravariable interactions.

In the encoding process, chemical features are first embedded into a high-dimensional space of dimension 128 using a dedicated module consisting of linear layers with normalization, activation, and dropout. This is followed by the addition of positional encodings to preserve level-specific information. A GNN layer, parameterized with embeddings of dimension 64, dynamically builds an adjacency matrix to represent relationships between pressure levels. Each of the three transformer layers combines GNN propagation for aggregating information across connected levels, ensuring that dependencies, such as vertical transport influences in atmospheric profiles, are captured, with self-attention mechanisms in the transformer component to extract information within each level.

This cooperation occurs iteratively across the three layers: The GNN component fuses interlevel information through matrix operations on the adjacency graph, whereas the transformer layers disseminate this aggregated knowledge via multihead attention, enhancing the representation of complex chemical interactions. This hybrid approach improves upon standalone transformer models by incorporating sparse, directed graph structures to robust handle hierarchical atmospheric data.

The multitask output head in the DRCAT model is structured to simultaneously predict OH concentrations and estimate associated uncertainties, inspired by principled multitask learning approaches that weigh losses based on homoscedastic uncertainty (*49*).

One critical challenge in developing satellite data–driven ML models is the nature of data itself. Although MLS measurements are generally considered ground truth, they are subject to instrument noise. Furthermore, MLS OH measurements are primarily available during a period of relative atmospheric chemical stability (2004 to 2009), limiting applicability to perturbed conditions, such as those following the 2022 Hunga eruption. Models trained exclusively on this dataset face two critical challenges: (i) the accuracy is restricted by the measurement noise (see section S1 for details), and (ii) they exhibit poor generalizability to unsampled conditions, like extreme events.

To mitigate these issues, DRCAT integrates a two-stage chemistry-informed training strategy, following a well-established “pretraining and fine-tuning” ML paradigm (*20*, *50*, *51*). In the initial pretraining stage, the model learns fundamental chemical knowledge from the enhanced SSA dataset. This enhanced SSA dataset incorporates random perturbations to simulate a comprehensive range of atmospheric states. The training target (SSA-OH) is calculated using a chemical box model that incorporates outputs from TOMCAT, with key species constrained by MLS observations, providing a low-noise, physically consistent OH representation. It should be noted that the pretrained model shares the same encoder structure with the main model other than the output layers. In the following stage, we initialize DRCAT from pretrained weights and train DRCAT on 2-year MLS observational dataset using a reduced learning rate for fine-tuning. After completion of training, DRCAT can reconstruct a continuous OH global dataset from 2004 to the present using available MLS measurements of input species.

Therefore, by adopting this framework, DRCAT is not limited by the scarcity of MLS OH observations and the biases of the SSA and CTM outputs. Instead, it learns the general chemical relations from the SSA dataset and correct the final OH predictions using the available MLS observations, resulting in a model that surpasses both SSA and CTM simulations.

### Loss function

A loss function is used to measure the model’s output and update the model’s parameters. Different loss functions penalize different aspects and can guide the model in different directions during the training process, which in return will affect the model’s performance.

Given that OH measurement noise varies with pressure, a standard mean squared error loss is insufficient as it would not account for this heteroscedasticity. We therefore adopt a heteroscedastic negative log-likelihood loss. The loss function L jointly optimizes OH concentration estimates and their uncertainties (*49*)

$$L\left(Y,\hat{Y},{\hat{\sigma }}^{2}\right)=\frac{1}{N_{p}}\sum_{p=1}^{N_{p}}\;\left[\underset{\text{precision}-\text{weighted error}}{\underbrace{\frac{1}{2}\text{exp}\left(-\text{log}{\hat{\sigma }}_{p}^{2}\right){\left(Y_{p}-{\hat{Y}}_{p}\right)}^{2}}}+\underset{\text{uncertainty regularizer}}{\underbrace{\frac{1}{2}\text{log}{\hat{\sigma }}_{p}^{2}}}\right]$$ (1)

where $N_{p}$ represent the number of pressure levels, ${\hat{\sigma }}_{p}^{2}$ represent the ML-predicted variance of OH at the pressure level p, and $Y_{p}$ and ${\hat{Y}}_{p}$ are the OH target labels and predictions at the pressure level p.

The first term scales prediction errors by the inverse of predicted variance, imposing stronger penalties when the model is confident but inaccurate. The second term prevents degenerate solutions by penalizing excessive uncertainty. By minimizing this composite loss, the model learns not only to make accurate predictions but also to provide a reliable estimate of its own uncertainty at each atmospheric level.

### Evaluation metric

To evaluate model performance fairly, we apply the global zonal average of OH data to avoid large noise in single vertical profiles. For global zonal OH concentration comparison, we compute metrics of RMSE, *R*^2^, and SSIM to quantitatively assess accuracy. These metrics are defined as follows

$$\text{RMSE}=\sqrt{\frac{1}{N_{\text{lat}}\cdot N_{\text{pressure}}}\sum_{i=1}^{N_{\text{lat}}}\;\;\sum_{j=1}^{N_{\text{pressure}}}\;\;{\left(y_{i,j}-{\hat{y}}_{i,j}\right)}^{2}}$$ (2)

$$R^{2}=1-\frac{\sum_{i=1}^{N_{\text{lat}}}\;\;\sum_{j=1}^{N_{\text{pressure}}}\;\;{\left(y_{i,j}-{\hat{y}}_{i,j}\right)}^{2}}{\sum_{i=1}^{N_{\text{lat}}}\;\;\sum_{j=1}^{N_{\text{pressure}}}\;\;{\left(y_{i,j}-\bar{y}\right)}^{2}}$$ (3)

where $y_{i,j}$ is the observed OH concentration at latitude index i and pressure level index j, and ${\hat{y}}_{i,j}$ is the corresponding predicted value. Lower values indicate superior precision, with zero representing exact agreement across the entire grid. For *R*^2^, values closer to 1 show a better fit.

SSIM is a method to measure the similarity between two images. It considers changes in structural information, luminance, and contrast. In this context, we apply SSIM to the global zonal OH concentration treated as image-like data, where an SSIM value quantifies the overall similarity between the predicted and reference OH distributions

$$\text{SSIM}\left(x,y\right)=\frac{\left(2\mu_{x}\mu_{y}+C_{1}\right)\left(2\sigma_{xy}+C_{2}\right)}{\left(\mu_{x}^{2}+\mu_{y}^{2}+C_{1}\right)\left(\sigma_{x}^{2}+\sigma_{y}^{2}+C_{2}\right)}$$ (4)

Here, x and y represent the reference and estimated OH distributions. $\mu_{y}$ and $\mu_{\hat{y}}$ are the means of the respective matrices, $\sigma_{y}^{2}$ and $\sigma_{\hat{y}}^{2}$ are the variances, $\sigma_{y\hat{y}}$ is the covariance, and $C_{1}$ and $C_{2}$ are stabilization constants. SSIM ranges from −1 to 1, with 1 denoting perfect similarity in structure, luminance, and contrast between the observed and estimated OH.

### Model training

The model parameters are optimized using the AdamW algorithm, an extension of the Adam optimizer that improves weight decay regularization, making it highly effective for training deep neural networks (*52*). Regularization incorporates dropout at a rate of 0.05 throughout the network to mitigate overfitting. Batch sizes are configured as 2048 not only for efficient GPU utilization but also for mitigating the data noise. The learning rate starts at the initial setting value and decreases over epochs. Different learning rates are applied for two training stages to optimize convergence: The first stage uses a higher initial learning rate of 0.001 to facilitate rapid learning of core chemical patterns, whereas the second stage uses a reduced initial learning rate of 0.0001 to enable fine-tuning.

Single training of DRCAT on three NVIDIA L40S 48 GB GPUs takes ~1 hour. After training completion, DRCAT can generate OH for the whole 20-year dataset on single GPU within 5 min. This inference speed is much faster than the CTM simulation and SSA method, which also requires simulated photochemical data.

## Supplementary Materials

### This PDF file includes:

Supplementary Text Figs. S1 to S12 Table S1 References
