# Supplementary material for: A chemistry-informed deep learning network for mitigating the stratospheric OH data gap
Source: Sci Adv. 2026 Jul 8;12(28):eaee2319. doi: 10.1126/sciadv.aee2319 (PMC13344375; doi:10.1126/sciadv.aee2319)
Supplement: Supplementary file 1 — Supplementary Text Figs. S1 to S12 Table S1 References [file sciadv.aee2319_sm.pdf]

Science Advances
AAAS

Supplementary Materials for

A chemistry-informed deep learning network for mitigating the stratospheric OH data gap

Wenjie Yin *et al.*

Corresponding author: Chen Zhou, chenzhou@whu.edu.cn; Martyn P. Chipperfield, M.Chipperfield@leeds.ac.uk

Sci. Adv. **12**, eaee2319 (2026)
DOI: 10.1126/sciadv.aee2319

This PDF file includes:

Supplementary Text
Figs. S1 to S12
Table S1
References

Supplementary Text

S1. Problem definition

The estimation of OH concentrations is critical for understanding atmospheric chemistry, yet complicated by inherent noise in Microwave Limb Sounder (MLS) observations. We aim to predict the OH vertical profiles from noisy satellite observations of key chemical species across a range of pressure levels.

The input observations derived from MLS can be represented as a set of multivariate vectors **X**:

$$\mathbf{X} = \{X_{i,t,p}\} \in \mathbb{R}^{N_c \times N_t \times N_p} \quad (1)$$

where:

$i \in \{O_3, H_2O, T, \ldots\}$ is the index for the chemical species (N_c total species).

t is the observation time index (N_t total observations).

p is the atmospheric pressure level index (N_p total levels).

The target variable, **Y**, is the observed OH concentration profile:

$$\mathbf{Y} = \{Y_{t,p}\} \in \mathbb{R}^{N_t \times N_p} \quad (2)$$

A fundamental challenge in this domain is that all satellite observations are subject to measurement error. We assume that the observed values are a combination of the true atmospheric state and an additive noise term.

Let $X^*_{i,t,p}$ and $Y^*_{t,p}$ represent the true input features and target. The observed values are then modelled as:

$$X_{i,t,p} = X^*_{i,t,p} + \epsilon_{x,i,p},\ \epsilon_{x,i,p} \sim \mathcal{N}(0, \sigma^2_{x,i,p}) \quad (3)$$

$$Y_{t,p} = Y^*_{t,p} + \epsilon_{y,p},\ \epsilon_{y,p} \sim \mathcal{N}(0, \sigma^2_{y,p}) \quad (4)$$

where ϵ_x and ϵ_y are the noise terms. We assume this noise is random, follows a Gaussian distribution with zero mean, and has a variance that may differ for each chemical species and pressure level. The pressure-dependent variance is particularly important, as instrument sensitivity often varies with altitude.

The goal is to learn a function f that takes the noisy input vector for a single observation time, $\mathbf{x}_t = \{X_{i,t,p}\}_{i,p}$, and produces an estimate, $\hat{\mathbf{y}}_t$, of the true OH profile, $\mathbf{y}^*_t = \{Y^*_{t,p}\}_p$.

$$\hat{\mathbf{y}}_t = f(\mathbf{x}_t; \theta) \quad (5)$$

where θ represents the model parameters.

Normally, ML model is trained by minimizing the expected mean squared error (MSE):

$$\arg\min_{\theta}\ \mathbb{E}\left[\frac{1}{\mathrm{T}}\sum_{t=1}^{\mathrm{T}} \left(f(\mathbf{X}_t; \theta) - Y_t\right)^2\right] \quad (6)$$

Under Gaussian noise, this formulation yields the Bayes-optimal estimator, which is the conditional expectation of the target given the inputs:

$$\hat{Y} = \mathbb{E}[Y|X] \quad (7)$$

In practice, the observed target **Y** is the sum of the true value Y^* and its noise term ϵ_Y.

$$\mathbb{E}[Y|X] = \mathbb{E}[(Y^* + \epsilon_Y)|X] \tag{8}$$

Assuming the measurement noise ϵ_Y is independent of the observations and has a mean of zero ($\mathbb{E}[\epsilon_Y] = 0$), we can simplify this expression:

$$\mathbb{E}[Y|X] = \mathbb{E}[Y^*|X] + \mathbb{E}[\epsilon_Y|X] = \mathbb{E}[Y^*|X] \tag{9}$$

This shows that the optimal estimator under MSE does not attempt to predict the noisy observation Y. Instead, it aims to predict the true OH concentration, Y^*, based on the noisy input observation **X**. The model learns to average the noise in the target variable.

However, the challenge of input noise remains. The estimator is conditioned on the noisy input X, not the true input X^*. Substituting the true physical relationship, $Y^* = f^*(X^*)$, we get:

$$\hat{Y} = \mathbb{E}[f^*(X^*)|(X^* + \epsilon_x)] \tag{10}$$

This expression reveals that the learned function acts as a smoothed version of $f^*(X^*)$. This estimate is a weighted average over all possible true input values X^*. The degree of this averaging will be affected by the noise variance in the input features **X**.

S2. Baseline methods

TOMCAT 3D Model

TOMCAT is a three-dimensional (3D) off-line chemical transport model (CTM) (*44*). The model uses winds and temperatures from meteorological analyses (e.g. from the European Centre for Medium-Range Weather Forecasts) to specify the atmospheric transport and temperatures and calculates the abundances of chemical species in the troposphere and stratosphere. The model incorporates a full chemical scheme that includes reactions relevant to OH production and loss. TOMCAT typically operates at resolutions ranging from ~2.8° × 2.8° to higher resolutions, depending on computational resources and study requirements. Vertical levels extend from the surface to the lower mesosphere, capturing variations of OH across a range of altitudes.

Observation-Constrained Steady State Approximation

The steady-state approximation (SSA) is a simplified method for estimating OH concentrations. Due to its short lifetime (~1s), OH concentrations are assumed to reach a balance between production and loss rates almost instantaneously, allowing the use of a steady-state equation to calculate its concentration (*48*):

$$[\text{OH}]_{\text{Steady State}} = \frac{k_{A+B}[A][B] + \cdots + j_C[C] + \cdots}{\sum k_D[D] + \cdots},$$

where the numerator represents a sum of the source terms. $k_{A+B}[A][B]$ is the reaction rate constant of A and B to form OH, and $j_C[C]$ is the photolysis coefficient of C to form OH. The denominator represents a sum of the sink terms. $k_D[D]$ is the reaction rate constant of D and OH, where D represents an individual sink species.

To apply this equation, concentrations of species and other parameters (e.g., H_2O, O_3, temperature, HNO_3) are derived from MLS observations. Other terms are derived from TOMCAT outputs. The performance of SSA method using satellite observations as constraints has been validated in the troposphere (*48*). This method simplifies the complex, time-dependent differential equations governing OH chemistry into an algebraic expression, making it computationally efficient for scientific use.

S3. Feature selection for DRCAT

The selection of appropriate features is a critical step in developing a robust and accurate ML model for predicting OH. The chosen features should exhibit chemical relations that influence OH concentration, while also considering data quality and alignment with model structure. This section details the process of identifying candidate features and the experiments for final feature selection.

Feature Selection Criteria

The OH radical plays a pivotal role in the stratosphere and is involved in reactions with many other chemical species. Features selected for the model are those with known chemical linkages to OH production and loss. The primary OH production pathway is the photolysis of O_3 in the presence of H_2O, yielding excited oxygen atoms ($O(^1D)$) that react to form OH via $O(^1D) + H_2O \rightarrow 2OH$. Key sinks include carbon monoxide (CO), methane (CH_4), and various volatile organic compounds (VOCs). Nitrogen oxides ($NO_x = NO + NO_2$) are also crucial as they are involved in both OH production (e.g., $HO_2 + NO \rightarrow OH + NO_2$) and loss cycles (e.g., $OH + NO_2 + M \rightarrow HNO_3 + M$). Those related chemical species observed by MLS are considered as prime candidates for feature selection here.

Given the focus on predicting OH vertical profiles and model architecture, DRCAT requires structured input features to provide consistent coverage. Therefore, MLS-observed species that do not span the full pressure range of interest are deemed unsuitable, as they introduce critical gaps in vertical resolution. For instance, MLS SO_2 covers 215-10 hPa and only significantly enhanced concentrations can be observed. In this case, SO_2 is not used due to the lack of SO_2 training samples and vertical gaps. However, HO_2, a member of the HO_x family, is included despite its measurement range starting at 22 hPa. As the gap at 32 hPa is minor, we add an input mask to the model thereby enabling DRCAT to process incomplete profiles robustly.

Feature Selection Experiments

To determine the optimal subset of features for the final model, we designed a series of feature selection experiments. Models trained on different feature sets are evaluated through the two-stage training scheme using the statistical metric of RMSE. The first stage utilized an SSA dataset to establish baseline chemical relationships, while the second stage incorporated MLS observational data to refine the model. As discussed in Section S1, the inherent noise in MLS OH data limits achievable RMSE improvements in the second stage due to inherent data uncertainties. Therefore, model performance in the first stage is also considered in feature importance experiments.

The feature importance scores presented in Fig. S12 represent the average improvement in test RMSEs across the two training stages using the feature reduction method (details are presented in Table S1). In this method, each feature is individually removed from the full feature set (baseline: H_2O, O_3, temperature, HNO_3, HO_2, HCl, N_2O), and the resulting change in RMSE is assessed, quantified by the importance score = RMSE after removal – baseline RMSE. The RMSEs from training stages 1 and 2 are summed with equal weights of 0.5. Additionally, the impact of adding CO, a biased species, was evaluated inversely, where importance is defined as the RMSE after addition minus baseline RMSE.

The feature reduction experiments reveal possible underlying OH patterns leaned by DRCAT, which align with stratospheric OH chemistry and MLS data characteristics. Notably, HNO_3 emerges as the most critical feature, with the largest increase in importance score upon removal. This reflects its dual role in OH chemistry: it serves as a primary sink via $OH + NO_2 + M \rightarrow HNO_3 + M$ and a source through photolysis ($HNO_3 + h\nu \rightarrow OH + NO_2$). N_2O also shows a positive importance score though the lowest possibly due to its long lifetime and slow conversion rate. N_2O serves as a key precursor in the NOx cycle via $N_2O + O(^1D) \rightarrow 2NO$. The importance of HNO_3 and N_2O indicates that DRCAT identifies the critical role of NOx in modulating OH balance in the middle and upper stratosphere.

H_2O is a major factor, driven by its role as the primary OH source via $O(^1D) + H_2O \rightarrow 2OH$. HCl exhibits moderate importance, which aligns with its role as a direct sink for OH through $OH + HCl \rightarrow H_2O + Cl$, releasing reactive chlorine that participates in catalytic ozone depletion cycles.

As it is involved in key HO_x interconversions (e.g., $HO_2 + NO \rightarrow OH + NO_2$), HO_2 demonstrates moderate importance score. Its importance might be underestimated due to data noise and day-night bias, which necessitates correction via day-minus-night differences, particularly at 22 hPa. Also, the input mask imposed on HO_2 input may also affect its performance. In contrast, adding CO, a key OH sink ($OH + CO \rightarrow CO_2 + H$), yields a negative score, indicating performance degradation. This negative impact is probably attributed to MLS CO biases (−70% to +50% in 32-1 hPa), which introduce noise that outweighs its chemical relevance. It underscores the need for bias correction or exclusion in noisy datasets when developing ML models.

It is important to note the limitations of the feature reduction method here if the information provided by the removed species overlaps with contributions from remaining features. In this case, its importance score may be underestimated, leading to a weakened ranking. In other words, while the scores offer valuable guidance for relative significance, they do not fully encapsulate the absolute contributions of these species to OH reactions in the middle and upper stratosphere.

Furthermore, we conducted an additional experiment (Exp-Spatiotemporal) by using time, latitude, longitude, and solar zenith angle (SZA), achieving a relatively high RMSE in the test MLS dataset. This is possibly due to OH being governed by solar-driven processes, i.e., OH production is primarily controlled by the photolysis of ozone. Hanisco et al. (*35*) also discovered a remarkably tight correlation of OH concentration with the SZA, which is also nearly invariant over latitudes ranging from 70° S to 90° N and all seasons in the lower stratosphere. Such a model (Exp-Spatiotemporal) learns a simplistic "shortcut" by modelling OH patterns using spatiotemporal information rather than capturing the underlying chemical dynamics. The reliance on historical spatiotemporal OH patterns lead to failure in predicting strongly perturbed OH spatiotemporal patterns under extreme conditions.

As a result, we exclude spatiotemporal information from input features to focus on a pure chemical model, relying on species (H_2O, O_3, temperature, etc.) to capture the fundamental chemical processes driving OH variability. As shown in Table S1, the full chemical feature set achieves highest precision and better representation of mutual chemical interactions. Notably, DRCAT with core species (e.g., H_2O, O_3, temperature, HNO_3) is also able to produce acceptable OH concentrations from 2007-2009 testset results, which broadens the model's utility across observational dataset with limited chemical species.

Besides, using different combinations of input features also provide a potential way to qualitatively understand the chemical processes that influence OH variability. From Figs. S10 and S11, we can observe obvious OH enhancements of two DRCAT variants with different input features: one is with H_2O, O_3 and temperature, and the other is with H_2O, O_3, temperature and HNO_3. It is clear that after the Hunga volcanic eruption, the model with the core species (e.g., H_2O, O_3, temperature) shows stronger OH enhancements (up to 80×10^6 molec. cm^{-3} in peak absolute value in Fig. S10). Incorporating additional sink terms, such as HNO_3, attenuates this enhancement. This modulation is possibly attributed to the model's learned representation of balanced chemical reactions: in the core set, H_2O and O_3 primarily serve as OH sources, while added sinks like HNO_3 introduce counterbalancing loss mechanisms that refine the net HO_x budget under perturbed conditions.

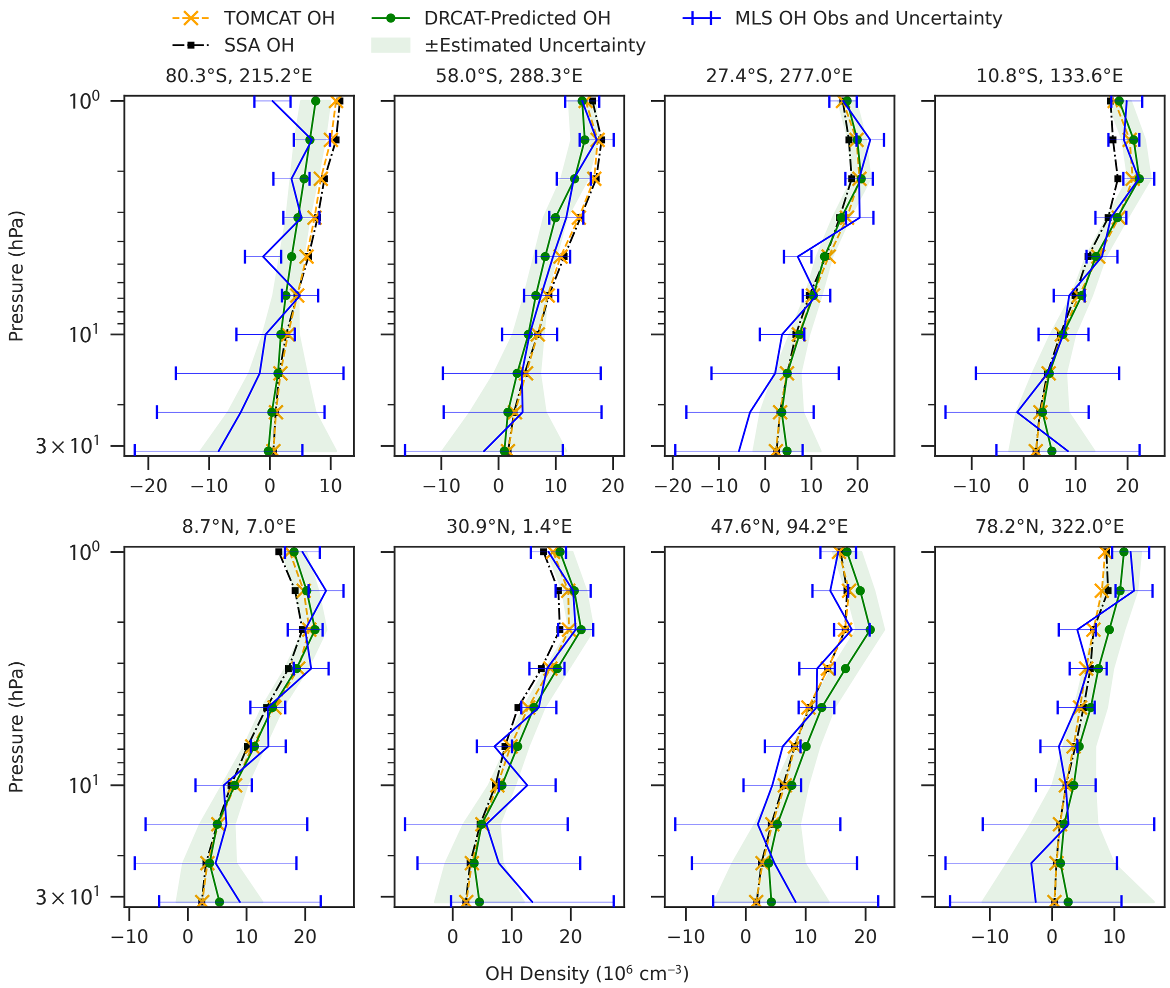

Fig. S1. Same as Figure 1 in main paper, with two additional OH data sources (TOMCAT and SSA OH).

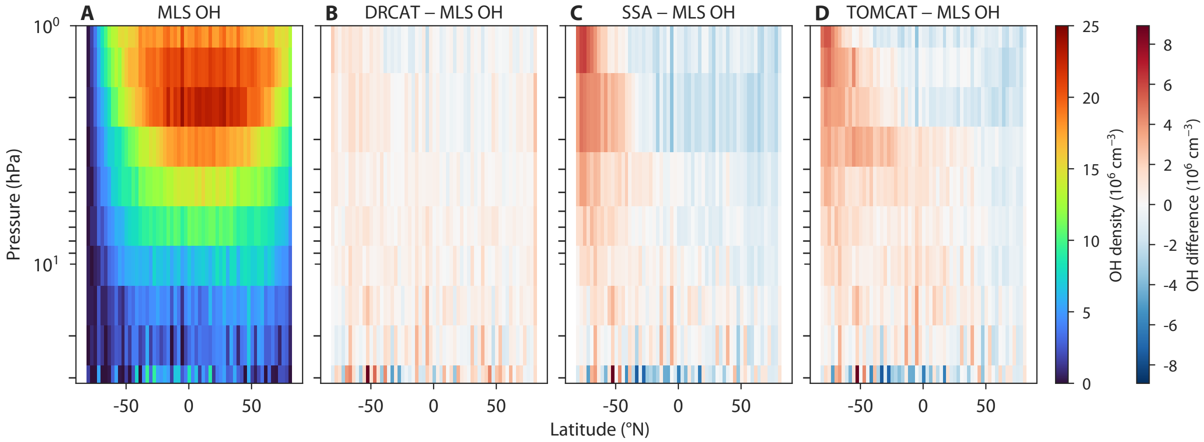

Fig. S2. Differences in global daily zonal mean OH on April 10, 2007, relative to MLS observations. (A) MLS OH observations. **(B)** DRCAT-predicted OH minus MLS OH observations. **(C)** Calculated SSA-OH minus MLS OH observations. **(D)** TOMCAT simulated OH minus MLS OH observations.

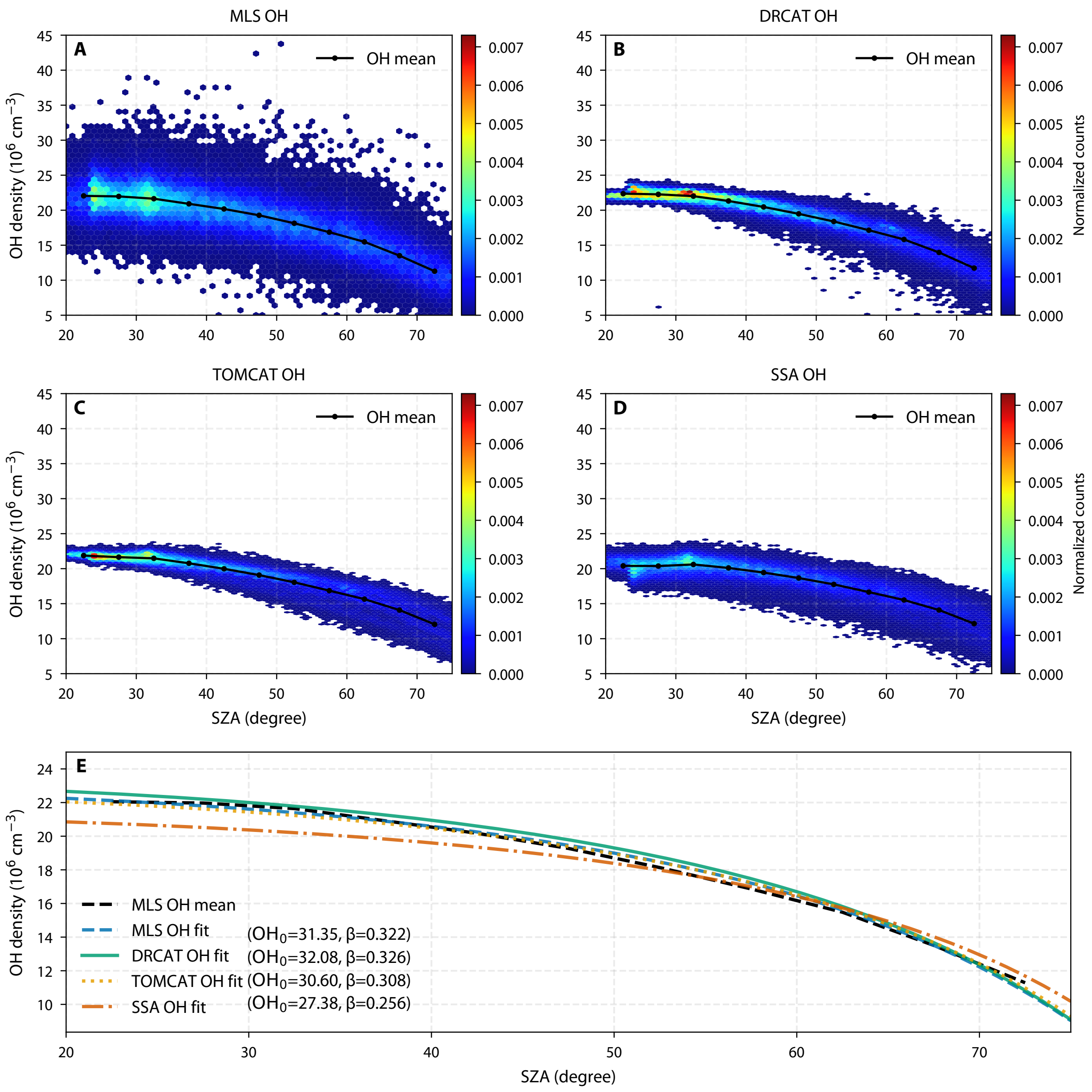

Fig. S3. OH diurnal variation as a function of solar zenith angle, at the 2.15 hPa pressure level during 2008. (**A-D**) Show the joint distribution of OH concentration and SZA for (A) MLS observations, (B) DRCAT OH, (C) TOMCAT OH, and (D) SSA OH, respectively. (**E**) Mean MLS OH curve and the corresponding two-parameter exponential fits for all three methods. Fit function: $OH = OH_0 exp(-\beta\, sec\,(SZA))$.

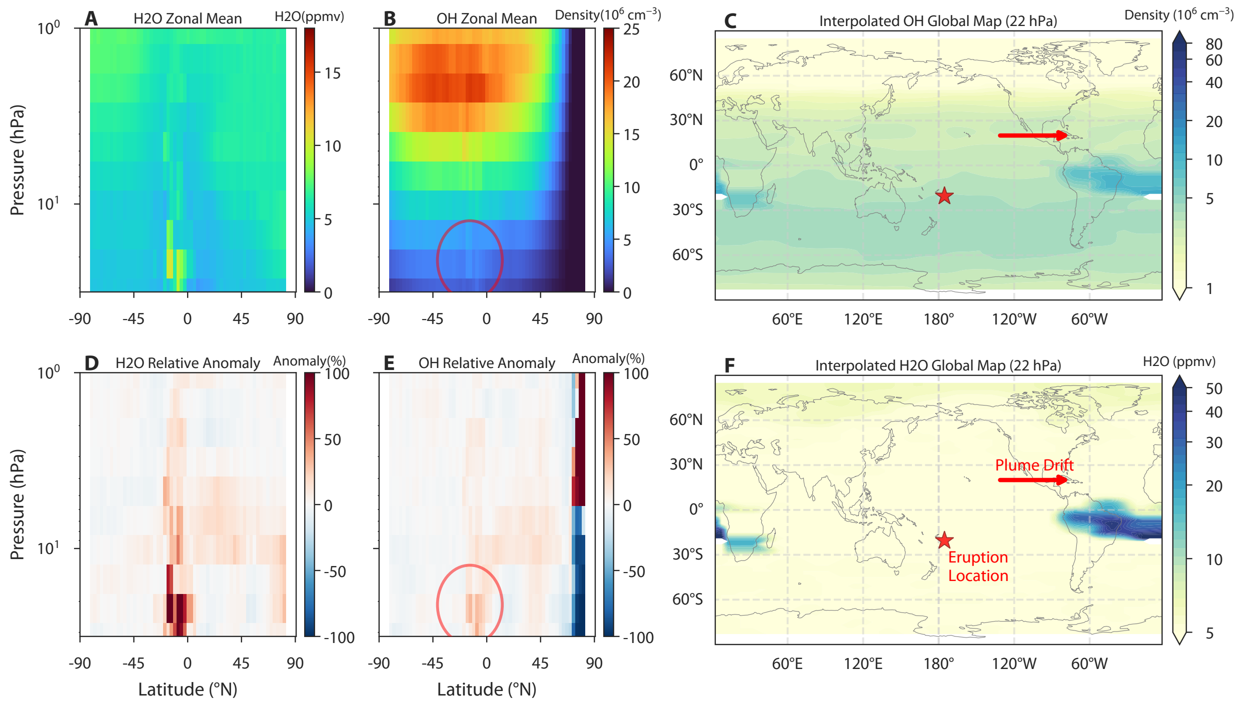

Fig. S4. As Figure 4 but for OH variations using calculated SSA.

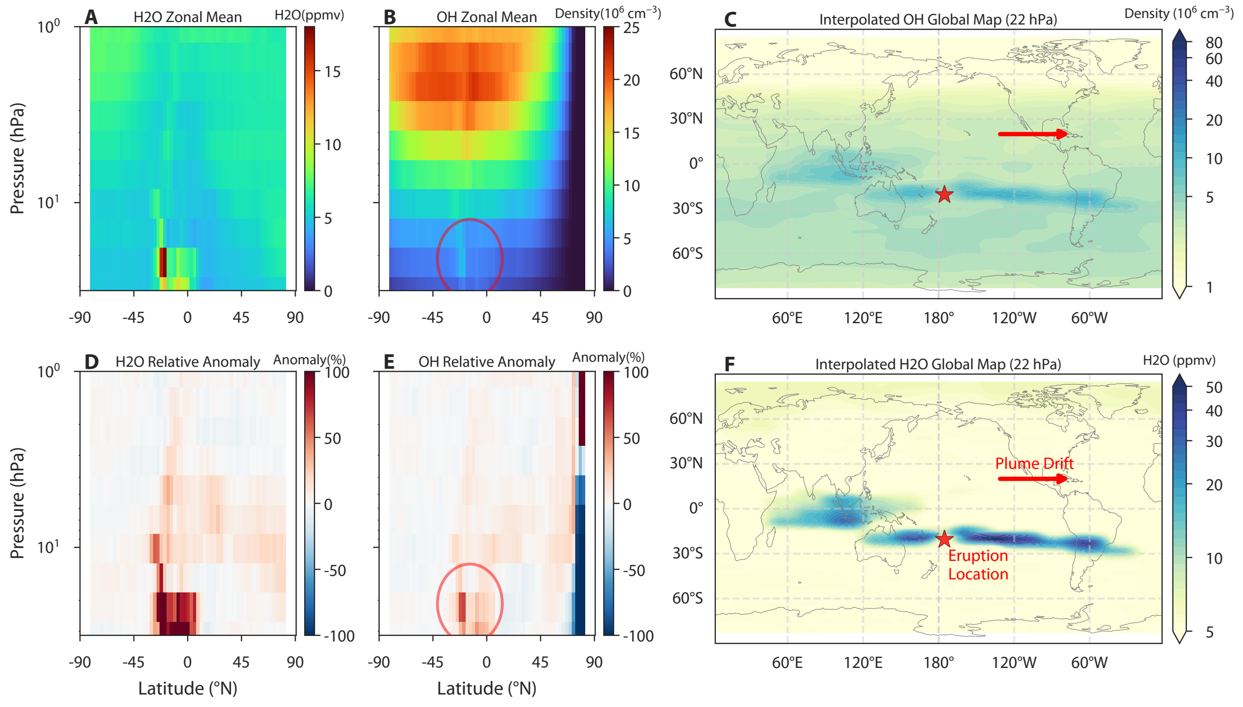

Fig. S5. As Figure 5 but for OH variations using calculated SSA.

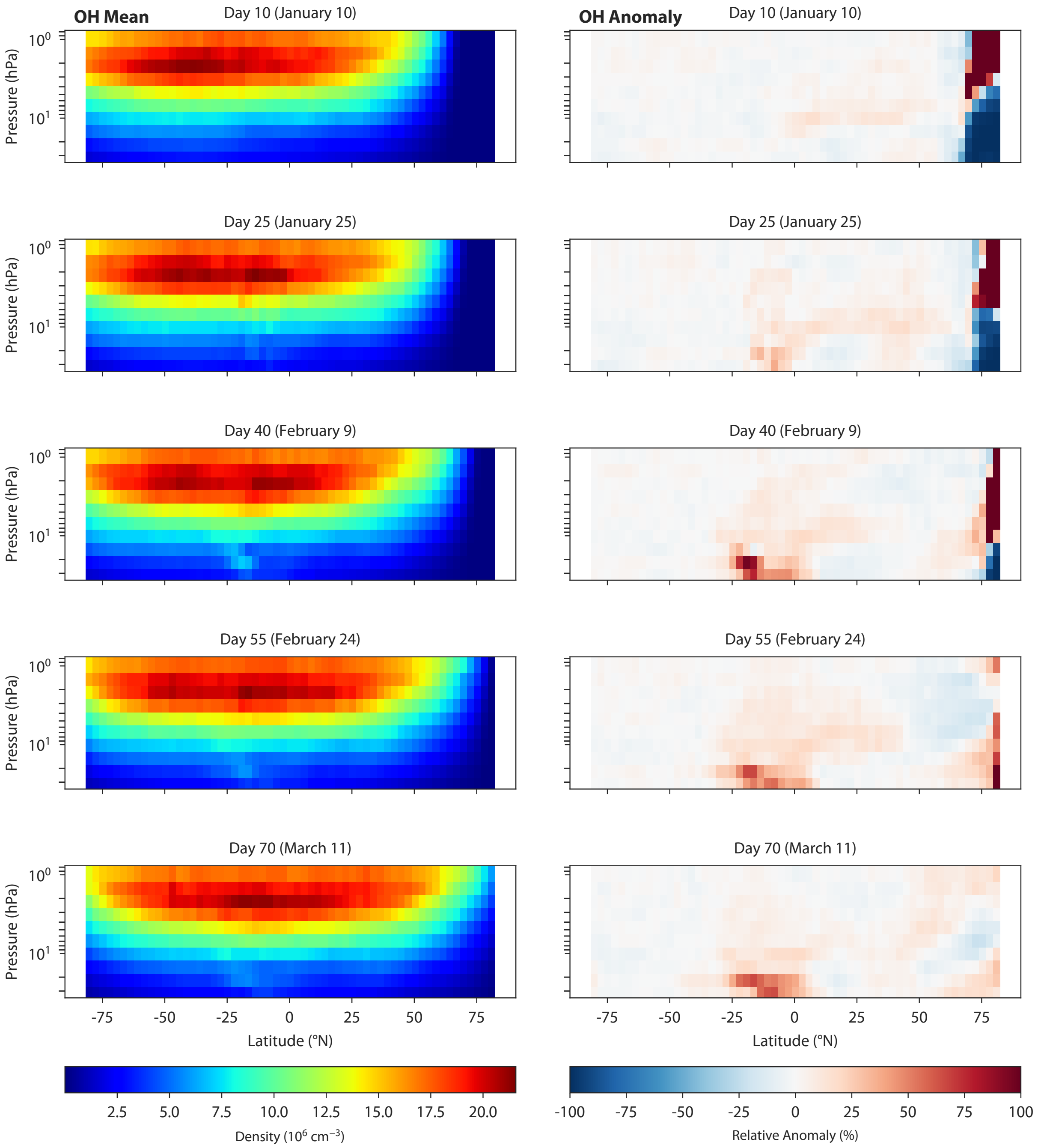

Fig. S6. SSA-calculated OH variations and OH relative anomaly (2022 vs 2021) from day 10 to 70 of the year, around the time of and following the Hunga eruption in early 2022.

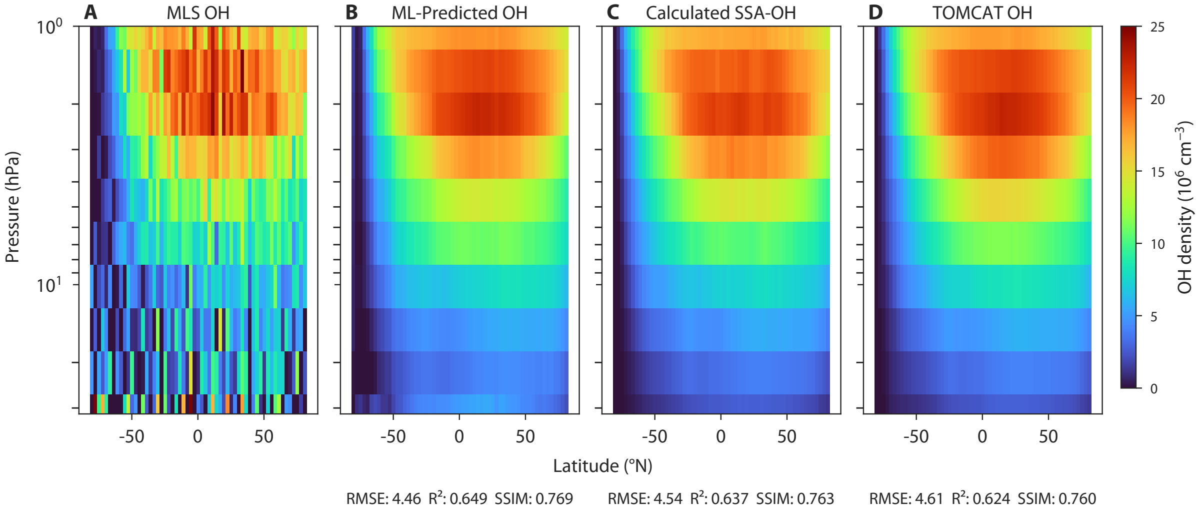

Fig. S7. Comparisons of global daily zonal mean OH for four data sources on August 16, 2011. (**A**) MLS OH observations. **(B)** DRCAT-predicted OH. **(C)** Calculated SSA-OH. **(D)** TOMCAT CTM simulation.

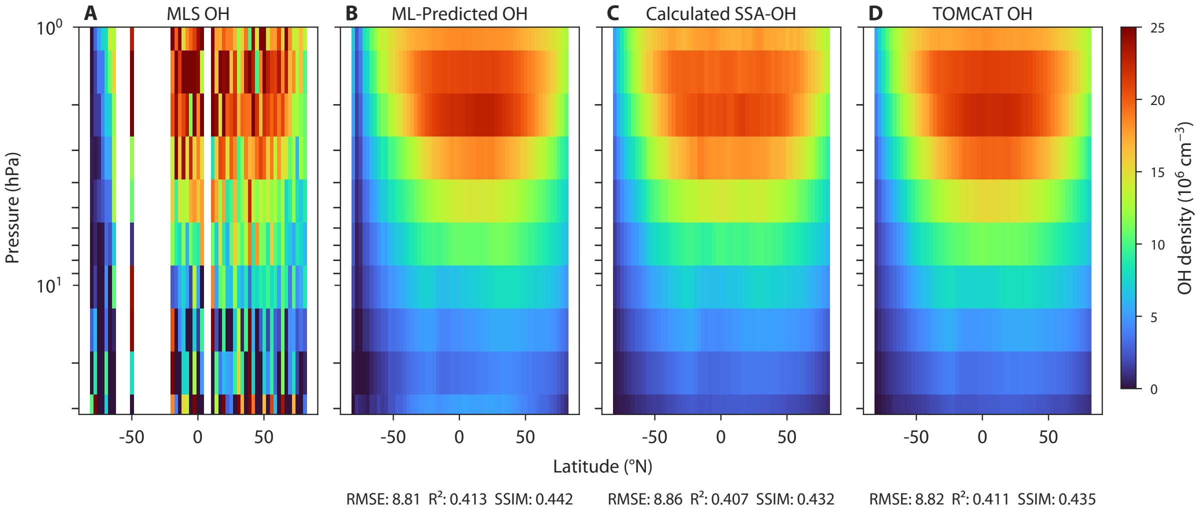

Fig. S8. Comparisons of global daily zonal mean OH for four data sources on September 5, 2014. (**A**) MLS OH observations. **(B)** DRCAT-predicted OH. **(C)** Calculated SSA-OH. **(D)** TOMCAT CTM simulation.

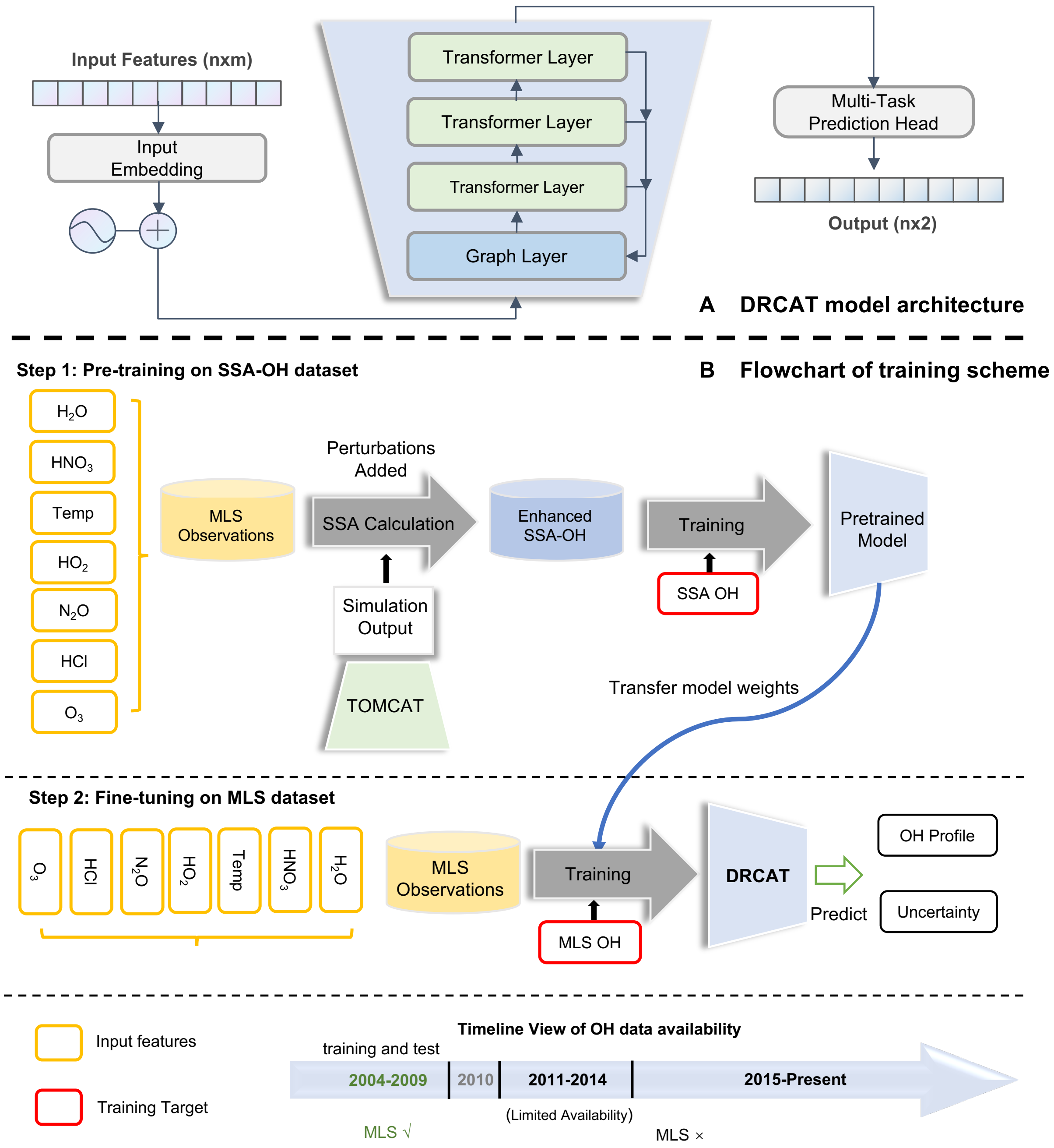

Fig. S9. Schematic diagram of DRCAT. (**A**) Detailed model architecture of DRCAT. (**B**) The flowchart of chemistry-informed two-stage model training phases (Some icons in this figure were adapted from (*53*), licensed under the MIT License).

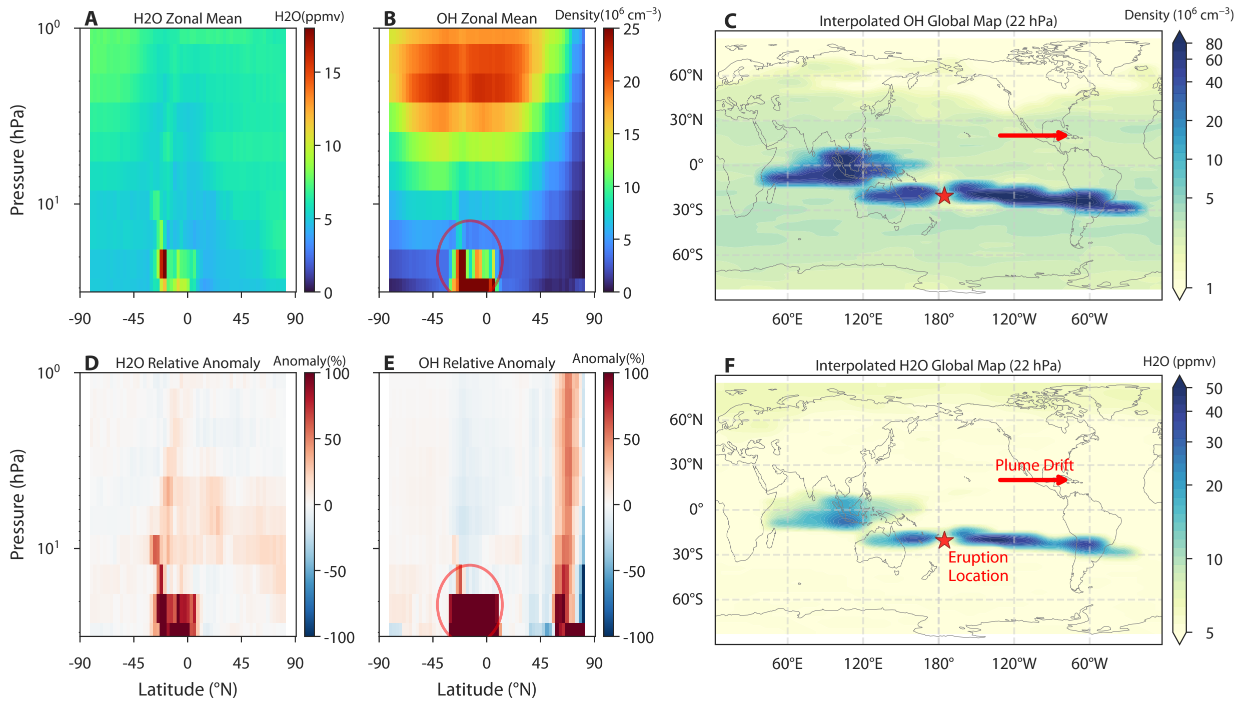

Fig. S10. As Figure 5 but for predicted OH variations using DRCAT with input features (H_2O, O_3, temperature).

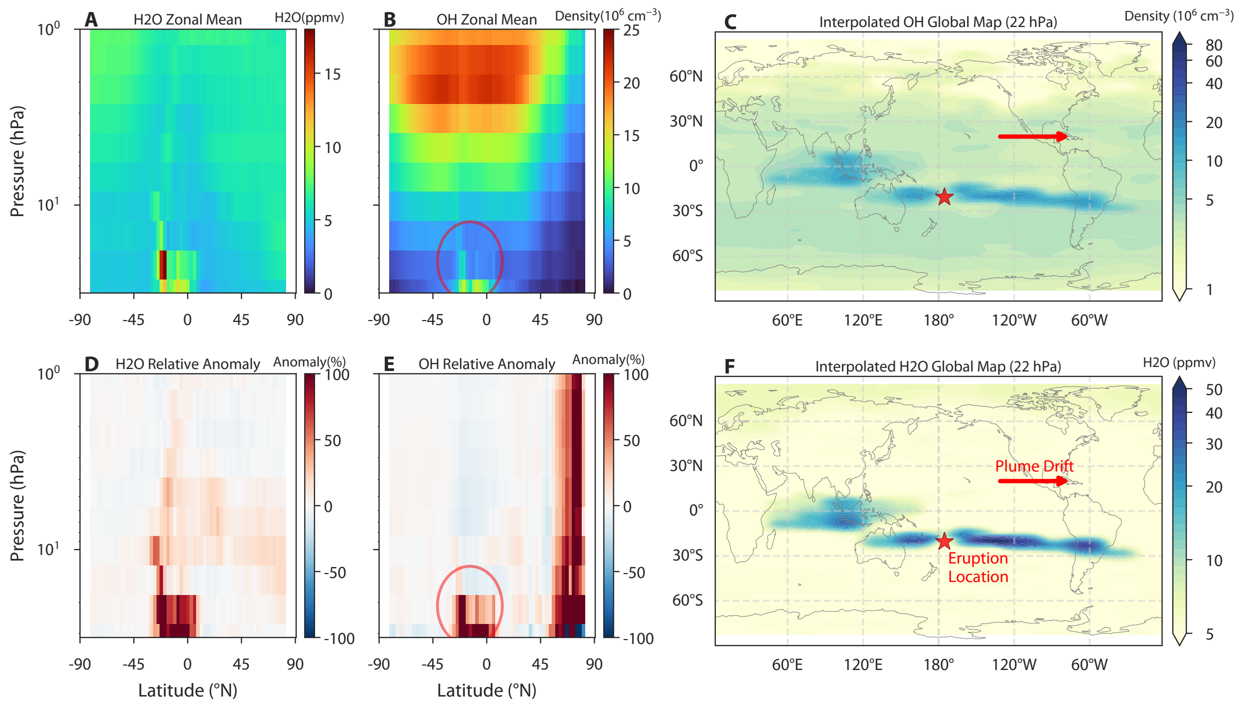

Fig. S11. As Figure 5 but for predicted OH variations using DRCAT with input features (H_2O, O_3, temperature, HNO_3).

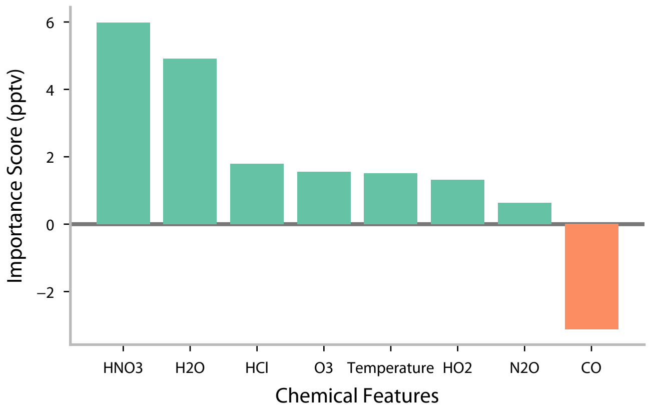

Fig. S12. Feature importance derived from DRCAT's feature removal experiments. (Note: importance score represents the average improvement of test RMSEs during two training stages relative to Exp-baseline. Units are in pptv, same as default MLS L2 data format.)

Table S1. The detailed results of models with different input feature sets.

Experiment name	Input species	Stage1 RMSE (pptv)	Stage2 RMSE (pptv)
Exp-baseline	H_2O, O_3, Temperature, HNO_3, N_2O, HCl, HO_2	7.58	31.86
Exp-H_2O	O_3, Temperature, HNO_3, N_2O, HCl, HO_2	16.84	32.47
Exp-O_3	H_2O, Temperature, HNO_3, N_2O, HCl, HO_2	11.51	32.44
Exp-Temperature	H_2O, O_3, HNO_3, N_2O, HCl, HO_2	9.92	32.54
Exp-HNO_3	H_2O, O_3, Temperature, N_2O, HCl, HO_2	17.68	33.73
Exp-N_2O	H_2O, O_3, Temperature, HNO_3, HCl, HO_2	8.45	32.26
Exp-HCl	H_2O, O_3, Temperature, HNO_3, N_2O, HO_2	10.44	32.59
Exp-HO_2	H_2O, O_3, Temperature, HNO_3, N_2O, HCl	9.41	32.67
Exp-CO	H_2O, O_3, Temperature, HNO_3, N_2O, HCl, HO_2, CO	13.55	32.13
Exp-Spatiotemporal	Day, Latitude, Longitude, SZA	12.16	32.14
Exp-4Species	H_2O, O_3, Temperature, HNO_3	9.15	32.18

REFERENCES

1. R. M. Stimpfle, P. O. Wennberg, L. B. Lapson, J. G. Anderson, Simultaneous, in situ measurements of OH and HO_2 in the stratosphere. *Geophys. Res. Lett.* **17**, 1905–1908 (1990).

2. W. H. Brune, I. C. Faloona, D. Tan, A. J. Weinheimer, T. Campos, B. A. Ridley, S. A. Vay, J. E. Collins, G. W. Sachse, L. Jaeglé, D. J. Jacob, Airborne in-situ OH and HO_2 observations in the cloud-free troposphere and lower stratosphere during SUCCESS. *Geophys. Res. Lett.* **25**, 1701–1704 (1998).

3. M. Carlotti, P. A. R. Ade, B. Carli, M. Chipperfield, P. A. Hamilton, F. Mencaraglia, I. G. Nolt, M. Ridolfi, Diurnal variability and night detection of stratospheric hydroxyl radical from far infrared emission measurements. *J. Atmos. Sol. Terr. Phys.* **63**, 1509–1518 (2001).

4. K. Chance, W. A. Traub, D. G. Johnson, K. W. Jucks, P. Ciarpallini, R. A. Stachnik, R. J. Salawitch, H. A. Michelsen, Simultaneous measurements of stratospheric HO_x, NO_x, and Cl_x: Comparison with a photochemical model. *J. Geophys. Res.* **101**, 9031–9043 (1996).

5. C. R. Burnett, E. B. Burnett, The regime of decreased OH vertical column abundances at Fritz Peak Observatory, CO: 1991–1995. *Geophys. Res. Lett.* **23**, 1925–1927 (1996).

6. F. P. Mills, R. P. Cageao, S. P. Sander, M. Allen, Y. L. Yung, E. E. Remsberg, J. M. Russell, U. Richter, OH column abundance over Table Mountain Facility, California: Intra-annual variations and comparisons to model predictions for 1997–2001. *J. Geophys. Res.* **108**, 2003JD003481 (2003).

7. H. M. Pickett, Microwave Limb Sounder THz module on Aura. *IEEE Trans. Geosci. Remote Sens.* **44**, 1122–1130 (2006).

8. J. W. Waters, L. Froidevaux, R. S. Harwood, R. F. Jarnot, H. M. Pickett, W. G. Read, P. H. Siegel, R. E. Cofield, M. J. Filipiak, D. A. Flower, J. R. Holden, G. K. Lau, N. J. Livesey, G. L. Manney, H. C. Pumphrey, M. L. Santee, D. L. Wu, D. T. Cuddy, R. R. Lay, M. S. Loo, V. S. Perun, M. J. Schwartz, P. C. Stek, R. P. Thurstans, M. A. Boyles, K. M. Chandra, M. C. Chavez, G.-S. Chen, B. V. Chudasama, R. Dodge, R. A. Fuller, M. A. Girard, J. H. Jiang, Y.

Jiang, B. W. Knosp, R. C. LaBelle, J. C. Lam, K. A. Lee, D. Miller, J. E. Oswald, N. C. Patel, D. M. Pukala, O. Quintero, D. M. Scaff, W. Van Snyder, M. C. Tope, P. A. Wagner, M. J. Walch, The Earth Observing System Microwave Limb Sounder (EOS MLS) on the Aura satellite. *IEEE Trans. Geosci. Remote Sens.* **44**, 1075–1092 (2006).

9. T. Canty, H. M. Pickett, R. J. Salawitch, K. W. Jucks, W. A. Traub, J. W. Waters, Stratospheric and mesospheric HO_x: Results from Aura MLS and FIRS-2. *Geophys. Res. Lett.* **33**, L12802 (2006).

10. H. M. Pickett, B. J. Drouin, T. Canty, R. J. Salawitch, R. A. Fuller, V. S. Perun, N. J. Livesey, J. W. Waters, R. A. Stachnik, S. P. Sander, W. A. Traub, K. W. Jucks, K. Minschwaner, Validation of Aura Microwave Limb Sounder OH and HO_2 measurements. *J. Geophys. Res.* **113**, D16S30 (2008).

11. S. Wang, H. M. Pickett, T. J. Pongetti, R. Cheung, Y. L. Yung, C. Shim, Q. Li, T. Canty, R. J. Salawitch, K. W. Jucks, B. Drouin, S. P. Sander, Validation of Aura Microwave Limb Sounder OH measurements with Fourier Transform Ultra-Violet Spectrometer total OH column measurements at Table Mountain, California. *J. Geophys. Res.* **113**, D22301 (2008).

12. S. A. Montzka, M. Krol, E. Dlugokencky, B. Hall, P. Jöckel, J. Lelieveld, Small interannual variability of global atmospheric hydroxyl. *Science* **331**, 67–69 (2011).

13. J. A. Pyle, A. M. Zavody, J. E. Harries, P. H. Moffat, Derivation of OH concentration from satellite infrared measurements of NO_2 and HNO_3. *Nature* **305**, 690–692 (1983).

14. J. A. Pyle, A. M. Závody, J. E. Harries, P. H. Moffat, Derivation of OH concentrations from LIMS measurements. *Adv. Space Res.* **4**, 117–120 (1984).

15. Q. Zhu, J. L. Laughner, R. C. Cohen, Combining machine learning and satellite observations to predict spatial and temporal variation of near surface OH in North American cities. *Environ. Sci. Technol.* **56**, 7362–7371 (2022).

16. D. C. Anderson, M. B. Follette-Cook, S. A. Strode, J. M. Nicely, J. Liu, P. D. Ivatt, B. N. Duncan, A machine learning methodology for the generation of a parameterization of the hydroxyl radical. *Geosci. Model Dev.* **15**, 6341–6358 (2022).

17. D. C. Anderson, B. N. Duncan, J. M. Nicely, J. Liu, S. A. Strode, M. B. Follette-Cook, Technical note: Constraining the hydroxyl (OH) radical in the tropics with satellite observations of its drivers—First steps toward assessing the feasibility of a global observation strategy. *Atmos. Chem. Phys.* **23**, 6319–6338 (2023).

18. Y. LeCun, Y. Bengio, G. Hinton, Deep learning. *Nature* **521**, 436–444 (2015).

19. A. Allen, S. Markou, W. Tebbutt, J. Requeima, W. P. Bruinsma, T. R. Andersson, M. Herzog, N. D. Lane, M. Chantry, J. S. Hosking, R. E. Turner, End-to-end data-driven weather prediction. *Nature* **641**, 1172–1179 (2025).

20. C. Bodnar, W. P. Bruinsma, A. Lucic, M. Stanley, A. Allen, J. Brandstetter, P. Garvan, M. Riechert, J. A. Weyn, H. Dong, J. K. Gupta, K. Thambiratnam, A. T. Archibald, C.-C. Wu, E. Heider, M. Welling, R. E. Turner, P. Perdikaris, A foundation model for the Earth system. *Nature* **641**, 1180–1187 (2025).

21. N. Bochow, A. Poltronieri, M. Rypdal, N. Boers, Reconstructing historical climate fields with deep learning. *Sci. Adv.* **11**, eadp0558 (2025).

22. F. Scarselli, M. Gori, A. C. Tsoi, M. Hagenbuchner, G. Monfardini, The graph neural network model. *IEEE Trans. Neural Netw.* **20**, 61–80 (2009).

23. A. Vaswani, N. Shazeer, N. Parmar, J. Uszkoreit, L. Jones, A. N. Gomez, Ł. Kaiser, I. Polosukhin, Attention is all you need. *Adv. Neural Inf. Proces. Syst.* **30**, 6000–6010 (2017).

24. Z. Zhang, L. Meng, Y. Gu, SageFormer: Series-aware framework for long-term multivariate time-series forecasting. *IEEE Internet Things J.* **11**, 18435–18448 (2024).

25. N. J. Livesey, W. G. Read, P. A. Wagner, L. Froidevaux, M. L. Santee, M. J. Schwartz, A. Lambert, L. F. Millan Valle, H. C. Pumphrey, G. L. Manney, “Earth Observing System

(EOS) Aura Microwave Limb Sounder (MLS) Version 5.0 x Level 2 and 3 Data Quality and Description Document, JPL D-105336 Rev. B" (Jet Propulsion Laboratory, 2022).

26. K. Minschwaner, G. L. Manney, S. H. Wang, R. S. Harwood, Hydroxyl in the stratosphere and mesosphere—Part 1: Diurnal variability. *Atmos. Chem. Phys.* **11**, 955–962 (2011).

27. L. Millán, M. L. Santee, A. Lambert, N. J. Livesey, F. Werner, M. J. Schwartz, H. C. Pumphrey, G. L. Manney, Y. Wang, H. Su, L. Wu, W. G. Read, L. Froidevaux, The Hunga Tonga-Hunga Ha'apai Hydration of the Stratosphere. *Geophys. Res. Lett.* **49**, e2022GL099381 (2022).

28. D. M. Wilmouth, F. F. Østerstrøm, J. B. Smith, J. G. Anderson, R. J. Salawitch, Impact of the Hunga Tonga volcanic eruption on stratospheric composition. *Proc. Natl. Acad. Sci. U.S.A.* **120**, e2301994120 (2023).

29. Y. Zhu, C. G. Bardeen, S. Tilmes, M. J. Mills, X. Wang, V. L. Harvey, G. Taha, D. Kinnison, R. W. Portmann, P. Yu, K. H. Rosenlof, M. Avery, C. Kloss, C. Li, A. S. Glanville, L. Millán, T. Deshler, N. Krotkov, O. B. Toon, Perturbations in stratospheric aerosol evolution due to the water-rich plume of the 2022 Hunga-Tonga eruption. *Commun. Earth Environ.* **3**, 248 (2022).

30. A. N. LeGrande, K. Tsigaridis, S. E. Bauer, Role of atmospheric chemistry in the climate impacts of stratospheric volcanic injections. *Nat. Geosci.* **9**, 652–655 (2016).

31. S. Rasp, S. Hoyer, A. Merose, I. Langmore, P. Battaglia, T. Russell, A. Sanchez-Gonzalez, V. Yang, R. Carver, S. Agrawal, M. Chantry, Z. Ben Bouallegue, P. Dueben, C. Bromberg, J. Sisk, L. Barrington, A. Bell, F. Sha, WeatherBench 2: A benchmark for the next generation of data-driven global weather models. *J. Adv. Model. Earth Syst.* **16**, e2023MS004019 (2024).

32. Y. Shi, R. Hu, N. Wu, H. Zhang, X. Liu, Z. Zeng, J. Zhu, P. Han, C. Luo, H. Zhang, J. He, X. Shi, Comparison of AI and NWP models in operational severe weather forecasting: A study on tropical cyclone predictions. *J. Geophys. Res. Mach. Learn. Comput.* **2**, e2024JH000481 (2025).

33. Y. Zheng, C. Lu, Z. Wu, J. Liao, Y. Zhang, Q. Wang, Machine learning-based model for real-time GNSS precipitable water vapor sensing. *Geophys. Res. Lett.* **49**, e2021GL096408 (2022).

34. R. Lam, A. Sanchez-Gonzalez, M. Willson, P. Wirnsberger, M. Fortunato, F. Alet, S. Ravuri, T. Ewalds, Z. Eaton-Rosen, W. Hu, A. Merose, S. Hoyer, G. Holland, O. Vinyals, J. Stott, A. Pritzel, S. Mohamed, P. Battaglia, Learning skillful medium-range global weather forecasting. *Science* **382**, 1416–1421 (2023).

35. T. F. Hanisco, E. J. Lanzendorf, P. O. Wennberg, K. K. Perkins, R. M. Stimpfle, P. B. Voss, J. G. Anderson, R. C. Cohen, D. W. Fahey, R. S. Gao, E. J. Hintsa, R. J. Salawitch, J. J. Margitan, C. T. McElroy, C. Midwinter, Sources, sinks, and the distribution of OH in the lower stratosphere. *J. Phys. Chem. A* **105**, 1543–1553 (2001).

36. T. Canty, K. Minschwaner, Seasonal and solar cycle variability of OH in the middle atmosphere. *J. Geophys. Res. Atmos.* **107**, 4737 (2002).

37. S. Wang, K.-F. Li, T. J. Pongetti, S. P. Sander, Y. L. Yung, M.-C. Liang, N. J. Livesey, M. L. Santee, J. W. Harder, M. Snow, F. P. Mills, Midlatitude atmospheric OH response to the most recent 11-y solar cycle. *Proc. Natl. Acad. Sci. U.S.A.* **110**, 2023–2028 (2013).

38. R. Geirhos, J.-H. Jacobsen, C. Michaelis, R. Zemel, W. Brendel, M. Bethge, F. A. Wichmann, Shortcut learning in deep neural networks. *Nat Mach. Intell.* **2**, 665–673 (2020).

39. A. Krizhevsky, I. Sutskever, G. E. Hinton, ImageNet classification with deep convolutional neural networks. *Commun. ACM* **60**, 84–90 (2017).

40. J. Schmidhuber, Deep learning in neural networks: An overview. *Neural Netw.* **61**, 85–117 (2015).

41. M. Li, E. Karu, C. Brenninkmeijer, H. Fischer, J. Lelieveld, J. Williams, Tropospheric OH and stratospheric OH and Cl concentrations determined from CH_4, CH_3Cl, and SF_6 measurements. *NPJ Clim. Atmos. Sci.* **1**, 29 (2018).

42. S. Rohs, C. Schiller, M. Riese, A. Engel, U. Schmidt, T. Wetter, I. Levin, T. Nakazawa, S. Aoki, Long-term changes of methane and hydrogen in the stratosphere in the period 1978–2003 and their impact on the abundance of stratospheric water vapor. *J. Geophys. Res.* **111**, D14315 (2006).

43. R. J. Salawitch, J. B. Smith, H. Selkirk, K. Wargan, M. P. Chipperfield, R. Hossaini, P. F. Levelt, N. J. Livesey, L. A. McBride, L. F. Millán, E. Moyer, M. L. Santee, M. R. Schoeberl, S. Solomon, K. Stone, H. M. Worden, The imminent data desert: The future of stratospheric monitoring in a rapidly changing world. *Bull. Am. Meteorol. Soc.* **106**, E540–E563 (2025).

44. M. P. Chipperfield, New version of the TOMCAT/SLIMCAT off-line chemical transport model: Intercomparison of stratospheric tracer experiments. *Q. J. R. Meteorol. Soc.* **132**, 1179–1203 (2006).

45. A. Chrysanthou, K. Dubé, S. Tegtmeier, M. P. Chipperfield, Hemispheric asymmetry in stratospheric trends of HCl and ozone: Impact of chemical feedback on ozone recovery. *J. Geophys. Res. Atmos.* **130**, e2024JD042161 (2025).

46. S. S. Dhomse, M. P. Chipperfield, W. Feng, R. Hossaini, G. W. Mann, M. L. Santee, Revisiting the hemispheric asymmetry in midlatitude ozone changes following the Mount Pinatubo eruption: A 3-D model study. *Geophys. Res. Lett.* **42**, 3038–3047 (2015).

47. S. S. Dhomse, M. P. Chipperfield, R. P. Damadeo, J. M. Zawodny, W. T. Ball, W. Feng, R. Hossaini, G. W. Mann, J. D. Haigh, On the ambiguous nature of the 11 year solar cycle signal in upper stratospheric ozone. *Geophys. Res. Lett.* **43**, 7241–7249 (2016).

48. M. A. Pimlott, R. J. Pope, B. J. Kerridge, B. G. Latter, D. S. Knappett, D. E. Heard, L. J. Ventress, R. Siddans, W. Feng, M. P. Chipperfield, Investigating the global OH radical distribution using steady-state approximations and satellite data. *Atmos. Chem. Phys.* **22**, 10467–10488 (2022).

49. R. Cipolla, Y. Gal, A. Kendall, “Multi-task learning using uncertainty to weigh losses for scene geometry and semantics” in *2018 IEEE/CVF Conference on Computer Vision and Pattern Recognition* (IEEE, 2018), pp. 7482–7491.

50. Y.-G. Ham, J.-H. Kim, J.-J. Luo, Deep learning for multi-year ENSO forecasts. *Nature* **573**, 568–572 (2019).

51. T. Brown, B. Mann, N. Ryder, M. Subbiah, J. D. Kaplan, P. Dhariwal, A. Neelakantan, P. Shyam, G. Sastry, A. Askell, Language models are few-shot learners. *Adv. Neural Inf. Proces. Syst.* **33**, 1877–1901 (2020).

52. I. Loshchilov, F. Hutter, Decoupled Weight Decay Regularization. arXiv:1711.05101 [cs.LG] (2017).

53. E. Saravia, ML Visuals (2021); https://github.com/dair-ai/ml-visuals.
